# Intra-annual fluctuation in morphology and microfibril angle of tracheids revealed by novel microscopy-based imaging

**DOI:** 10.1371/journal.pone.0277616

**Published:** 2022-11-15

**Authors:** Yusuke Kita, Tatsuya Awano, Arata Yoshinaga, Junji Sugiyama

**Affiliations:** Laboratory of Tree Cell Biology, Division of Forest and Biomaterials Science, Graduate School of Agriculture, Kyoto University, Kyoto, Japan; USDA Forest Service, UNITED STATES

## Abstract

Woody cells, such as tracheids, fibers, vessels, rays etc., have unique structural characteristics such as nano-scale ultrastructure represented by multilayers, microfibril angle (MFA), micro-scale anatomical properties and spatial arrangement. Simultaneous evaluation of the above indices is very important for their adequate quantification and extracting the effects of external stimuli from them. However, it is difficult in general to achieve the above only by traditional methodologies. To overcome the above point, a new methodological framework combining polarization optical microscopy, fluorescence microscopy, and image segmentation is proposed. The framework was tested to a model softwood species, *Chamaecyparis obtusa* for characterizing intra-annual transition of MFA and tracheid morphology in a radial file unit. According our result, this framework successfully traced the both characteristics tracheid by tracheid and revealed the high correlation (|*r*| > 0.5) between S_2_ microfibril angles and tracheidal morphology (lumen radial diameter, tangential wall thickness and cell wall occupancy). In addition, radial file based evaluation firstly revealed their complex transitional behavior in transition and latewood. The proposed framework has great potential as one of the unique tools to provide detailed insights into heterogeneity of intra and inter-cells in the wide field of view through the simultaneous evaluation of cells’ ultrastructure and morphological properties.

## Introduction

Woody cells, such as tracheids, fibers, vessels, rays etc., have unique characteristics represented by their nm-scale ultrastructure (e.g. multilayers named by primary and secondary cell wall [1,2], microfibril angle (MFA) in each layer [3,4]), anatomical properties (e.g. transverse cell geometry and cell length [5,6]) and spatial arrangements from anatomical viewpoints. These characteristics are largely different in softwoods or hardwoods, and affected by external environmental factors such as precipitation, solar radiation, drought and so on [7,8]. Ideally, all of the above points should be measured simultaneously for understanding their relationships and extracting the effects of external stimuli from their anatomy.

In the case of softwoods, the above-mentioned anatomical features can be characterized in different structural orders, mm, μm and nm order. Transitional behaviors within an annual ring in sub-mm or mm order is well known as the eye-observable anatomical characteristics and proxy as the external stimuli [9]. In μm order, clear distinguishment of different cell species is achievable. In addition, qualitive and quantitative cell anatomical characteristics (pit structures and types, cell wall thickness, cell transverse area, lumen diameter, etc. [5,10]) affecting wood density and hydraulic features [11,12] can be evaluated. The most representative index in nm order is S_2_-layer MFA, and a lot of methodology have been developed for measuring it (e.g. electron microscope observation, X-ray, optical microscope observation combined with some preprocessing, reviewed in [4]) because it largely influences wood mechanical properties and anisotropy [13,14]. These anatomical features can be considered as the multiplication of the species-specific and the result of optimization to external environment.

It is ideal to measure all the above points simultaneously in order to understand how the anatomical features from different scale relate to one another and to determine how external stimuli affect them. There are, however, a lot of technical difficulties to achieve the goal. A part of studies tackled this kind of problems [15,16], but their mutual relationships remain ambiguous in many aspects in softwoods, which have been extensively researched in each resolution separately.

Recently, attractive polarization optical microscopy (POM)-based techniques have been introduced for semiquantitative evaluation of MFA in plant cell walls from cell transverse sections [17–20]. This method is superior in obtaining not only MFA but also the transverse cell anatomical properties and cell spatial arrangement at the same time compared with other methods for measurement mentioned above. Combined with the image segmentation techniques [21], S_2_-layer MFA and anatomical parameters of tracheids can be simultaneously and systematically evaluated.

This paper pays attention to important quantitative anatomical characteristics in softwoods, cell anatomical properties and cell spatial arrangement in transverse dimension and S_2_-layer MFA, and proposes a new methodological framework combining POM, fluorescence microscopy (FLM), and image segmentation to simultaneously measure and quantify the above-mentioned indices. *Chamaecyparis obtusa* was selected by a model softwood specimen for validating our methodology. As the next step, we aim to quantify intra-annual transition of tracheids’ transverse dimension, spatial arrangement and S_2_-layer MFA in a simultaneous manner as the application of our methodology. Especially, quantification of tracheids’ special arrangement is conducted by tracheidograms [22] considering the way of cell generation in softwoods [23].

## Materials & methods

### Sample preparation

A normal wood block of *C*. *obtusa* sampled from the mature heartwood region was cut into small wood sticks containing one annual ring and embedded in Spurr resin (Spurr Low Viscosity Embedding Kit, Polysciences, Warrington, PA, USA). Transverse sections of 5 μm thickness were cut with a rotary microtome (Sorvall JB-4, DuPont de Numours, Inc., Wilmington, DE, USA) and a diamond knife (HistoJumbo, Diatome Ltd, Helmastrasse, Switzerland). The sections were placed on a glass slide and dehydrated on a hot plate. Finally, they were sealed with a cover glass and a drop of Bioleit mounting agent (Okenshoji Co., Ltd., Tokyo, Japan).

### Experimental procedure after sample preparation (Fig 1)

**Fig 1:**
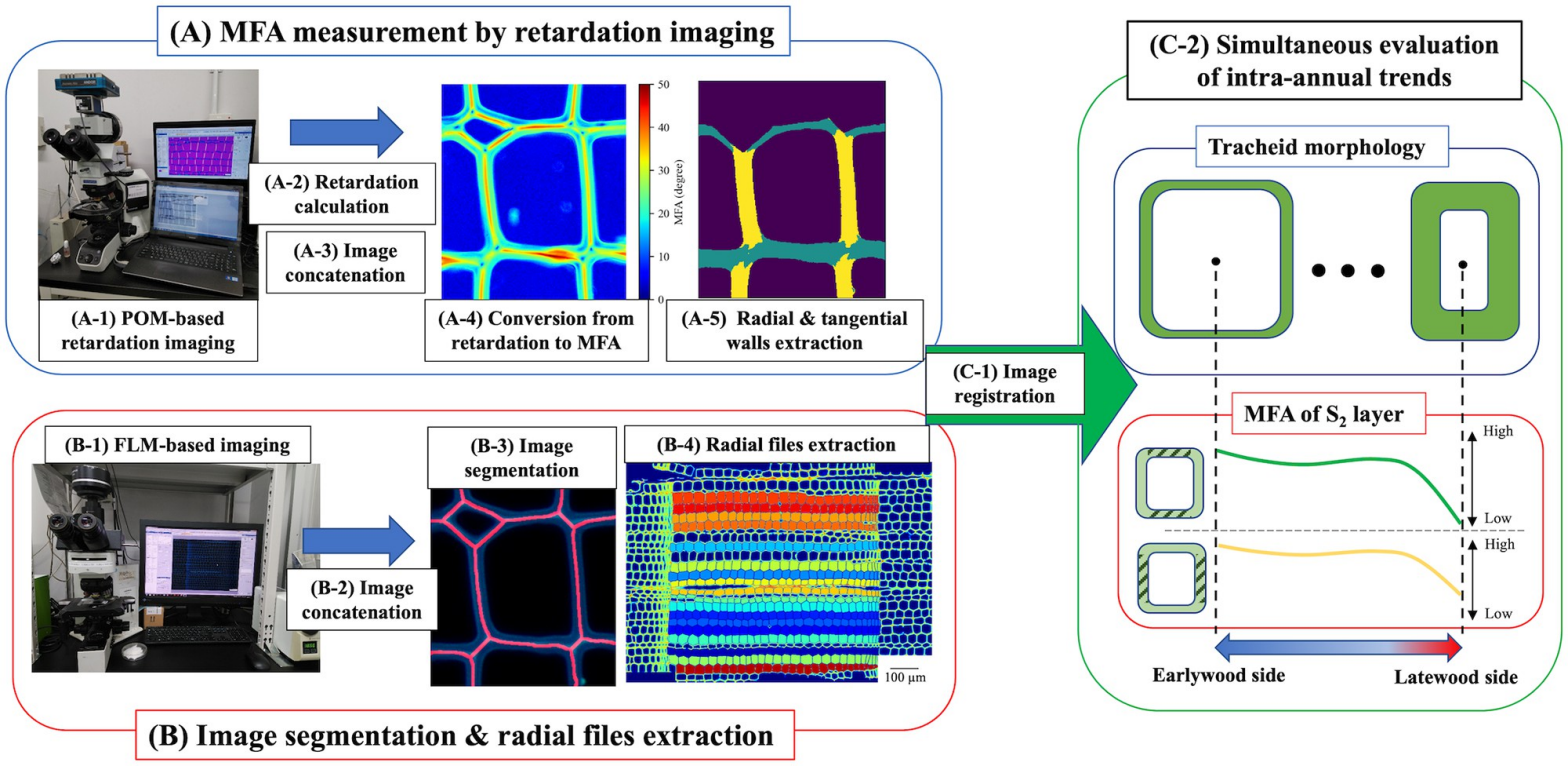
Experimental flows for image detection and analysis. The S_2_ microfibril angle (MFA) values of radial and tangential walls were obtained from polarization optical microscopy (POM)-based imaging (step A). Tracheid morphology in a radial file unit was extracted from fluorescence microscopy (FLM)-based imaging and image analyses (step B). Finally, the two results were integrated to simultaneously evaluate S_2_ MFA and tracheid morphology (step C). Details of each experimental step are explained in the main text.

An overview of the experimental procedure after sample preparation is presented in Fig 1. The procedure can be subdivided into three steps, designated A, B, and C. In step A (Fig 1, upper left), POM-based retardation images of the transverse section were captured and concatenated to generate a single image, as done in panoramic photography. The retardation image was converted to an MFA image. Pixels of radial and tangential cell walls were separately obtained by using azimuthal angle distributions. In step B (Fig 1, lower left), FLM-based images of the same section were captured and concatenated in the same manner. Cell segmentation and radial files extraction were applied to the fluorescence micrographs. In step C (Fig 1, right), the POM-based and FLM-based results were combined to obtain the tracheid anatomy and S_2_ MFA on radial and tangential walls in a radial files unit. Details of each experimental step are described in the following sections. Experimental procedures, such as image processing, calculations of retardation, MFA by POM image and MFA calculation from X-ray diffraction diagrams, were conducted using the programs written in Python language (https://github.com/pywood21/po_mfa_2022).

### POM and FLM measurement (Fig 1A-1 and 1B-1)

**Fig 2:**
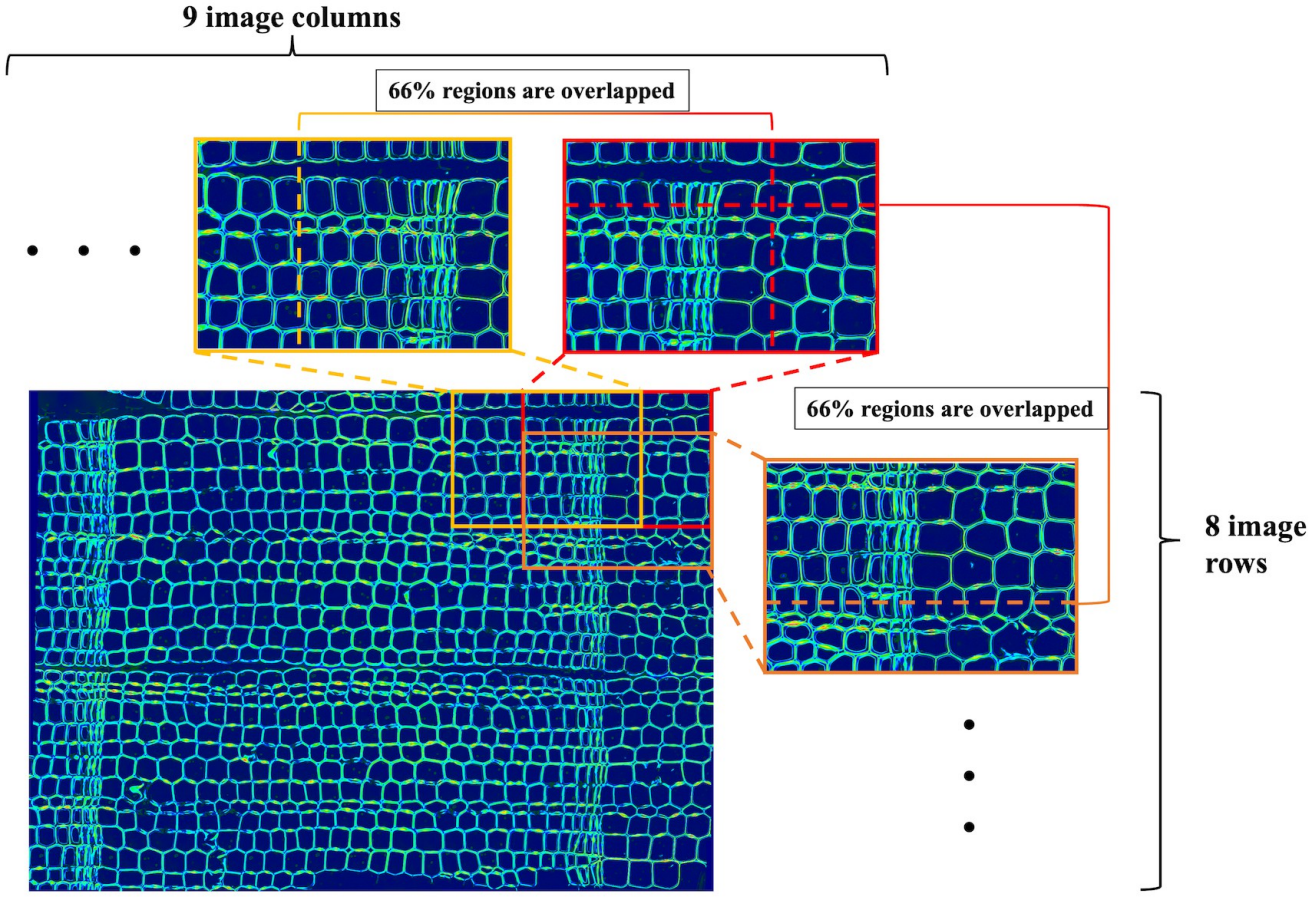
Example of image concatenation applied to a set of retardation images. The reconstructed retardation image comprised 72 partly overlapping retardation images in total (9 columns × 8 rows). Each image contained 66% overlapping regions with adjacent image patches in the horizontal and vertical directions.

All polarization optical micrographs of the transverse section were captured with a polarization optical microscope (BX51-P, Olympus Corporation, Tokyo, Japan) equipped with a ×40 magnification objective lens (UPLFLN40XP, NA = 0.75, Olympus Corporation) and a quarter-wavelength retardation plate (UTP-137, Olympus Corporation). Image detection was conducted with a front-illuminated monochrome CCD camera (Alta F2, Apogee Imaging Systems, Inc., Roseville, CA, USA) connected to a liquid crystal tunable bandpass filter (VariSpec^™^, VIS: 400–720 nm, 7 nm bandwidth; Cambridge Research & Instrumentation, Inc., Woburn, MA, USA). The wavelength center of the bandpass and exposure time were set to 546 nm and 1 s, respectively. Three images per spot were captured under different polarization states by rotating a polarizer [24]. In total, 72 images of 1536 pixels × 1024 pixels (9 columns × 8 rows, see Fig 2) of the section were set as imaging targets for capturing micrographs of the entire sectional region. Each adjacent image partially overlapped (approximately 66%) for the following image concatenation step. In addition, background images were captured in the same manner for background correction [24].

Fluorescence micrographs of the same section were captured under two fluorescence excitation/emission conditions using the fluorescent filter cubes U-MWU2 and U-MWIG3 (Olympus Corporation). The former cube is for detecting ultraviolet (UV)-excited autofluorescence from lignin aromatic skeletons, and the latter is for selectively detecting the green-light-excited fluorescence from ray-deposits, respectively. Measurements of fluorescence micrographs were performed using a BX50 microscope equipped with a U-HGLGPS fluorescence illumination source (Olympus Corporation) and ×10 magnification objective lens (UPLANFL, NA = 0.30, Olympus Corporation). All images were detected using a RGB color CCD camera (DP72, Olympus Corporation). In total, four images (4140 pixels × 3096 pixels) were captured to cover the entire region used for POM measurement. Each adjacent image partially overlapped for the above-mentioned reason.

#### Conversion of polarization optical micrographs (Fig 1A-2)

The pairs of optical micrographs captured under the three polarization states were converted to retardation and azimuthal angle images using the ‘three-frame algorithm without extinction setting’ originally proposed by [24].

#### Image concatenation (Fig 1A-3 and 1B-2)

Patches of the POM-based images (retardation, azimuthal angle, and MFA) and fluorescent light micrographs were concatenated to reconstruct the entire imaging regions (Fig 2). Concatenations of each image were achieved using the phase-only correlation method [25].

#### Conversion of retardation image to MFA image (Fig 1A-4)

The MFA image was obtained by the procedure as described in [13,16]. The values of ordinary and extraordinary refraction indices of cellulose fibers (*n*_o_ = 1.529 and *n*_e_ = 1.599, respectively) were extracted from [26]. In our study, the cellulose ratio of tracheids was tentatively set to 50% corresponding to the most abundant S_2_ layer following general trend seen in softwoods [27] (the cellulose content of not only S_2_ layer but of all layers correspond to 40–45% [28]). In the conversion step, net cellulose difference between earlywood and latewood was ignored because it (several percent at most in the case of *Picea abies* [29]) doesn’t largely affect the result of the conversion step.

#### Radial and tangential S_2_ MFA extraction (Fig 1A-5)

**Fig 3:**
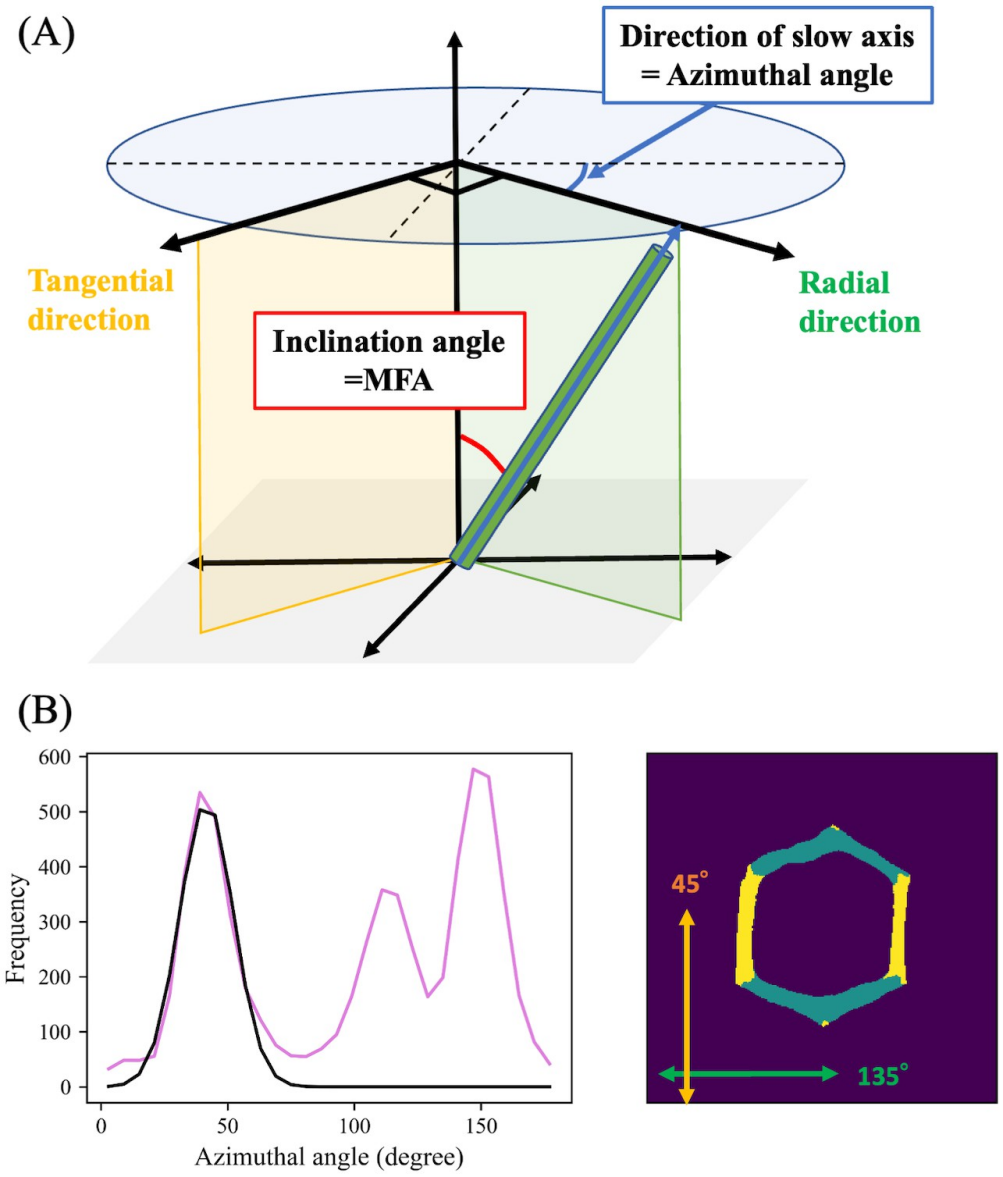
Azimuthal angle calculation from retardation imaging, and selective radial and tangential wall extraction from azimuthal angle distributions. (A) Definitions of azimuthal angle, and radial and tangential directions in the present experimental condition. Radial and tangential planes are colored green and yellow, respectively. (B) Azimuthal angle distribution (left, purple line) of a hexagonal-shaped tracheid (right). Tangential and radial walls are defined as pixels whose azimuthal angle is near to 45° (left: Area covered by the black curve; right: Yellow pixels, directions of yellow double-headed arrows) or the remaining parts (right: Green pixels).

The MFA values of radial and tangential walls were separately obtained by utilizing azimuthal angle distributions (see Fig 3) similar to those obtained by X-ray experiments [30]. Under the experimental conditions, pixels of tangential walls mainly contributed to peaks appearing at approximately 45°. The corresponding azimuthal angles were fitted by a single Gaussian function. Pixels whose azimuthal angles were within three *σ* from the peak center of the fitted Gaussian function, *μ*, were set as tangential wall components. The remaining parts were automatically set to radial wall components.

To separate MFA contributions of minor layers (S_1_ and S_3_) and of the S_2_ layer, selective S_2_ or S_1_+S_3_ layer detection was performed using a local valley or ridge detection algorithm implemented in the Python package “scikit-image” [31].

#### Segmentation and manual correction (Fig 1B-3)

Ray regions were subtracted from the concatenated UV-excited fluorescence micrographs to only evaluate tracheid anatomy. Ray regions were selectively extracted from the green-light-excited fluorescence micrographs.

After the above preprocessing step, cell boundary detection based on a watershed algorithm [32] was applied to the binarized image of the concatenated UV-excited fluorescence micrographs. This segmentation step was performed using the Python package “Mahotas” [33]. The watershed algorithm worked well for earlywood but generally did not for latewood because of the lack of a lumen in latewood tracheids. Therefore, cell boundaries were checked visually and manually corrected, especially in the latewood portion. After the segmentation steps, only the tracheid transverse area was measured in preparation for the following radial files extraction step.

#### Radial files and anatomical parameter extraction (Fig 1B-4)

The radial files extraction technique originally proposed by [34] was applied to the fluorescence micrograph after watershed segmentation. Briefly, this technique can extract radial files by utilizing graph networks termed region adjacency graphs [35] and the selection algorithm of the most probable adjacent tracheids based on their relative positions and morphological similarities (Bray–Curtis criterion [36]). In the selection algorithm, the optimal relative positions were controlled by the angle restriction, *θ* ≤ 25, defined by the angle between a horizontal vector and vector connecting two centroids of adjacent tracheids. The tracheid transverse area was used to calculate the Bray–Curtis criterion. In this step, 16 intact radial files were successfully detected.

After radial file extraction, anatomical parameters [37] (lumen transverse area, cell wall area, cell wall occupancy, tracheid and lumen radial diameters, tracheid tangential diameter, and tangential wall thickness) of tracheids belonging to the 16 radial files were measured. Measurement of tangential wall thickness was conducted based on the method of [38]. In addition, tracheid and lumen radial diameters, tracheid tangential diameter, and tangential wall thickness of approximately 100 tracheids were manually measured using ImageJ [39] for validation of the automatic measurement results (see S1 Fig).

#### Integration of POM and FLM-based data (Fig 1C-1)

Image registration of the reconstructed POM-based and FLM-based images was performed with SIFT [40]. Through this step, information extracted from the POM and FLM was matched.

#### Correlation analysis, radial file normalization and ANOVA analysis (Fig 1C-2)

Correlation coefficients (*r*) between tracheid morphology and mean S_2_ MFA on radial or tangential walls of 432 tracheids were calculated using Pearson correlation analysis after elimination of outliers in tangential wall thickness (S2 Fig). The screening was applied to tangential wall thickness after radial file normalization (described below) and the results obtained were reflected to the raw data.

For qualitative evaluation of intra-annual trends based on radial files, tracheid numbers of each radial file were normalized to the mean tracheid number (27 tracheids per radial file [8]). Each radial file array of mean S_2_ MFA was smoothed using the Savitzky–Golay filter (window size = 3, polynomial order = 1 [41]) for denoising.

After normalizing radial files and applying them to Shapiro-Wilk test and Bartlett test for checking normality and homoscedasticity of their distribution, multiple comparison with Steel-Dwass test was conducted to compare differences of each anatomical parameter depending on tracheids’ positions in tracheidograms.

#### X-ray diffraction analysis

For validating result of POM experiment, X-ray diffraction (XRD) -based MFA measurement was conducted to the same specimen. XRD diagrams of the resin embedded wood block were recorded on a cylindrical image plate using a RAXIS RAPID II (Rigaku Corporation, Tokyo, Japan). The graphite monochromated Cu Ka radiation generated at 50 kV and 100 mA was collimated to 0.3 mm*φ* and incident perpendicular to the longitudinal axis of the sample. Diffraction patterns were recorded from two spots, earlywood and latewood. Their MFA values were calculated by Cave’s method [42] using diffraction patterns from (200) plane.

## Results

### Retardation imaging and S_2_ MFA selective detection

**Fig 4:**
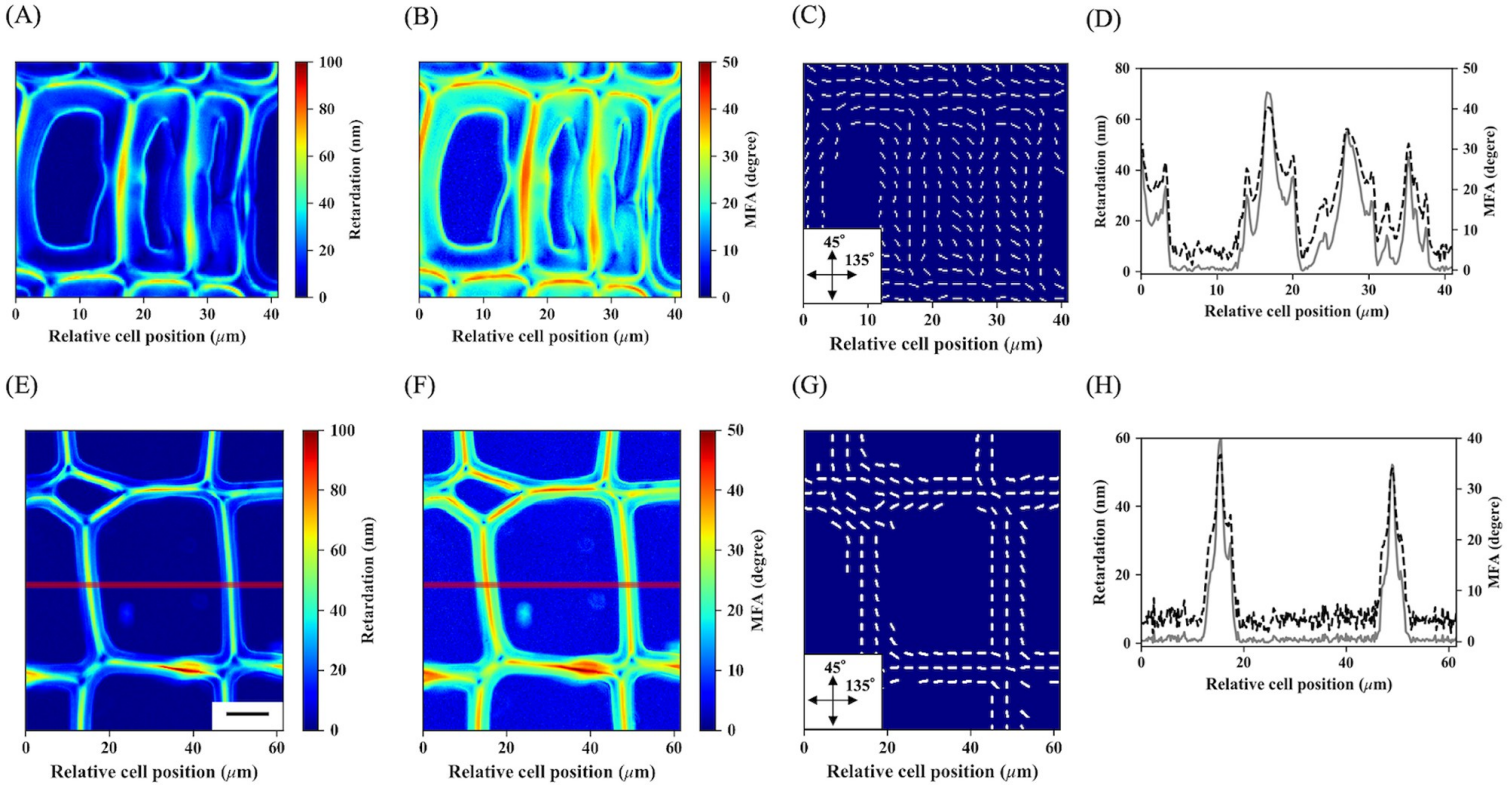
Examples of retardation, microfibril angle (MFA), and azimuthal angle images. (A–C) Retardation, MFA, and azimuthal angle images of latewood tracheids. (E–G) those of earlywood tracheids. (D) and (H) Sectional views of retardation (grey solid line) and MFA values (black dotted line) along red lines drawn on latewood tracheids (A and B) and earlywood tracheids (E and F). In (D) and (H), the S_2_ and S_1_+S_3_ layers appear as local-intensity minima and maxima, respectively. Bars, 10 μm.

Fig 4 shows the retardation, MFA, and azimuthal images obtained for latewood and earlywood tracheids. In Fig 4A, 4B, 4E and 4F, multi-layers consisting of the main S_2_ and minor S_1_ and S_3_ layers can be discriminated by their different retardation and MFA values. Fig 4D and 4H shows that the S_2_ in earlywood and latewood, S_1_, and S_3_ layers appear as local-intensity minima or maxima, and the MFA values correspond to approximately 20°, 10–15°, 30–40°, and 20–30°, respectively. The S_2_ MFA values recorded seem to be slightly higher because of the experimental imperfection of the MFA conversion step (see Discussion), but are roughly consistent with those of normal wood of *C*. *obtusa* reported in previous work [43–45]. The MFA values of the S_1_ and S_3_ layers largely contradict those reported by direct observation with transmission electron microscopy [46], because these layers are too thin to be resolvable by microscopic techniques using visible-light regions [47], in addition to the difference in their chemical compositions [27]. As a result, contributions of the minor two layers are merged with those of the S_2_ layer nearest to them and show extremely low MFA values.

**Fig 5:**
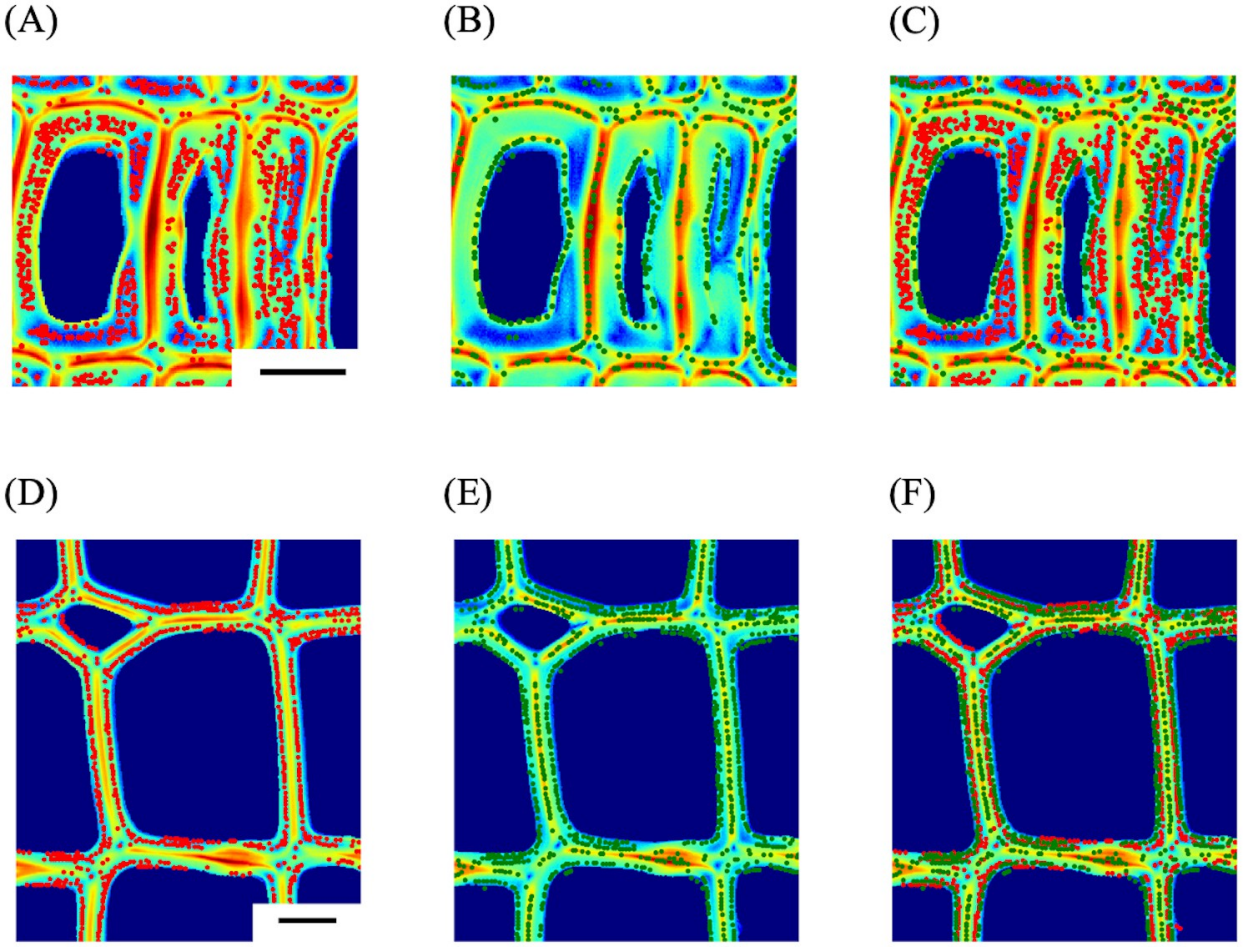
Selective detection of S_2_ or S_1_+S_3_ layers. (A) S_2_ detection and (B) S_1_+S_3_ detection in a latewood, (D) S_2_ detection and (E) S_1_+S3 detection in an earlywood. (C) and (F) are merged images of (A) and (B), and (D) and (E), respectively. Red and green dots correspond to the detected S_2_ (local-intensity minima) and S_1_+S_3_ (local-intensity maxima) layers’ contributions, respectively. Bars, 10 μm.

Fig 5 shows the results of selective S_2_ or S_1_ and S_3_ layer detections corresponding to the same tracheids shown in Fig 4. Local-intensity minima and maxima detection worked well as selective detections of the S_2_ (red dots) and S_1_+S_3_ layers (green dots) both in latewood and earlywood. Considering the lateral resolution of the ×40 objective lens (NA = 0.75) [48], and the cell wall thickness and ratio of each layer in tracheids of *C*. *obtusa* [47], pixels corresponding to the local-intensity minima can be treated as the pure contribution of the S_2_ layer.

### Intra-annual trends of tracheid morphology

**Fig 6:**
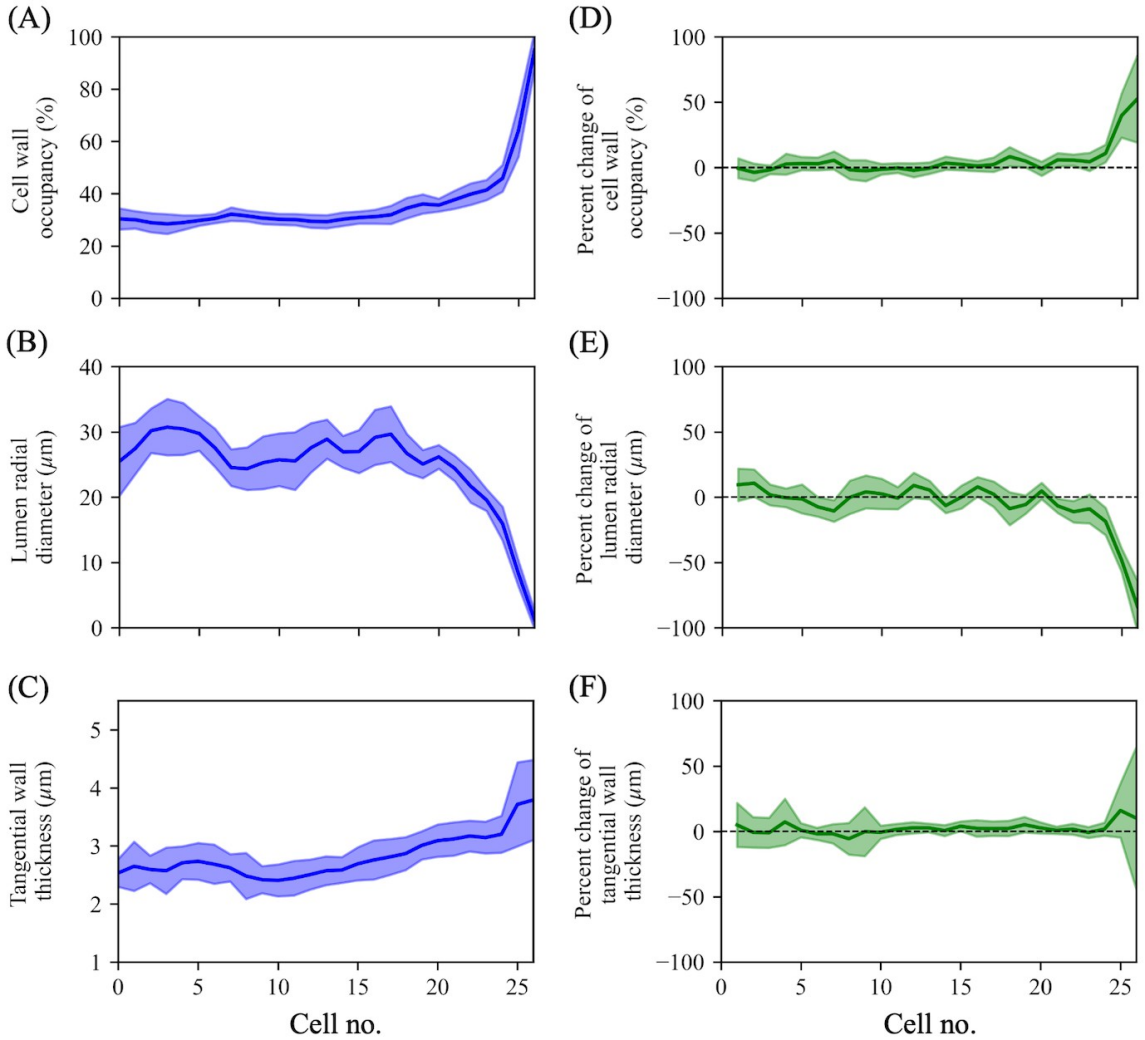
Normalized intra-annual transitional behaviors of a portion of the anatomical parameters. (A) Cell wall occupancy, (B) lumen radial diameter, and (C) tangential wall thickness, respectively. (D–F) Percentage changes of each anatomical parameter (A–C) from preceding tracheids. Solid lines and the shaded area surrounding them in each figure indicate the mean intra-annual transitions and their standard deviations, respectively. Cell no. 1 corresponds to the tracheid positioned at the most earlywood. Cell number ranges from no. 1 to no. 27 in normalized radial files.

Fig 6 shows intra-annual transitional behaviors of cell wall occupancy, radial lumen diameter, and tangential wall thickness. The remaining anatomical parameters are shown in S4 Fig. The anatomical parameters can be roughly classified into two groups: those without or with distinct intra-annual changes. The former is represented by cell wall area (S4C Fig) and tracheid tangential diameter (S4E Fig), and the remaining parameters can be regarded as examples of the latter. In the former, cell wall area is almost constant, but accompanied with slight decrement in the middle earlywood and the latewood (S4C Fig). Among the latter, parameters characterizing cell expansion (tracheid and lumen transverse area, S4A and S4B Fig; tracheid and lumen radial diameter, Fig 6D and S4D Fig) tend to show radical transitions (large percentage changes; see Fig 6E, S4F, S4G and S4I Fig) compared with those relating to secondary cell wall deposition (tangential wall thickness) [49]. Cell wall occupancy is affected both by contributions of cell expansion and secondary wall deposition. Its radical increment mainly follows the tendency seen in the parameters relating to cell expansion.

Standard deviations of tangential wall thickness of only the last tracheids are considerably larger than those of the other tracheids (see Fig 6C and 6F) because a portion of their walls tends to be thin compared with tracheids next to the last, as observed in other softwoods [50].

### Simultaneous evaluation of intra-annual trends of tracheid morphology and S_2_ MFA

Table 1 lists the correlation coefficients between tracheid morphology and mean S_2_ MFA on radial or tangential walls of 432 tracheid cells. Anatomical parameters are clearly divided into two groups: those with or without moderate correlation with S_2_ MFA. The former (*r* > 0.500, *P* < 0.05) are represented by lumen transverse area (radial wall: *r* = 0.408, *P* < 0.05; tangential wall: *r* = 0.512, *P* < 0.05), lumen radial diameter (radial wall: *r* = 0.473, *P* < 0.05; tangential wall: *r* = 0.517, *P* < 0.05), tangential wall thickness (radial wall: *r* = −0.452, *P* < 0.01; tangential wall: *r* = −0.555, *P* < 0.05), and cell wall occupancy (radial wall: *r* = −0.519, *P* < 0.05; tangential wall: *r* = −0.554, *P* < 0.05). Examples of the latter are cell wall area (radial wall: *r* = −0.003, *P* > 0.05; tangential wall: *r* = 0.059, *P* > 0.05) and tracheid tangential diameter (radial wall: *r* = 0.032, *P* > 0.05; tangential wall: *r* = 0.181, *P* < 0.05). These tendencies match entirely those observed in the preceding section. As a general trend, mean S_2_ MFA on tangential walls shows a stronger correlation with the tracheid anatomical parameters and a lower standard deviation than S_2_ MFA on radial walls because of the effects of MFA heterogeneity induced by pits only on the radial walls [51,52].

**Table 1:** Correlation coefficients between anatomical parameters and S_2_ microfibril angle (MFA).

Mean S_2_ MFA	Anatomical parameters							
	Tracheid transverse area	Tracheid radial diameter	Tracheid tangential diameter	Lumen transverse area	Lumen radial diameter	Cell wall area	Cell wall occupancy	Tangential wall thickness
Radial wall	**0.360**	**0.448**	0.032	**0.408**	**0.473**	-0.003	**-0.519**	**-0.452**
Tangential wall	**0.462**	**0.482**	**0.181**	**0.512**	**0.517**	0.059	**-0.554**	**-0.555**

Statistically significant values (*P* < 0.05) are indicated in bold text. Observed cell number is 432.

**Fig 7:**
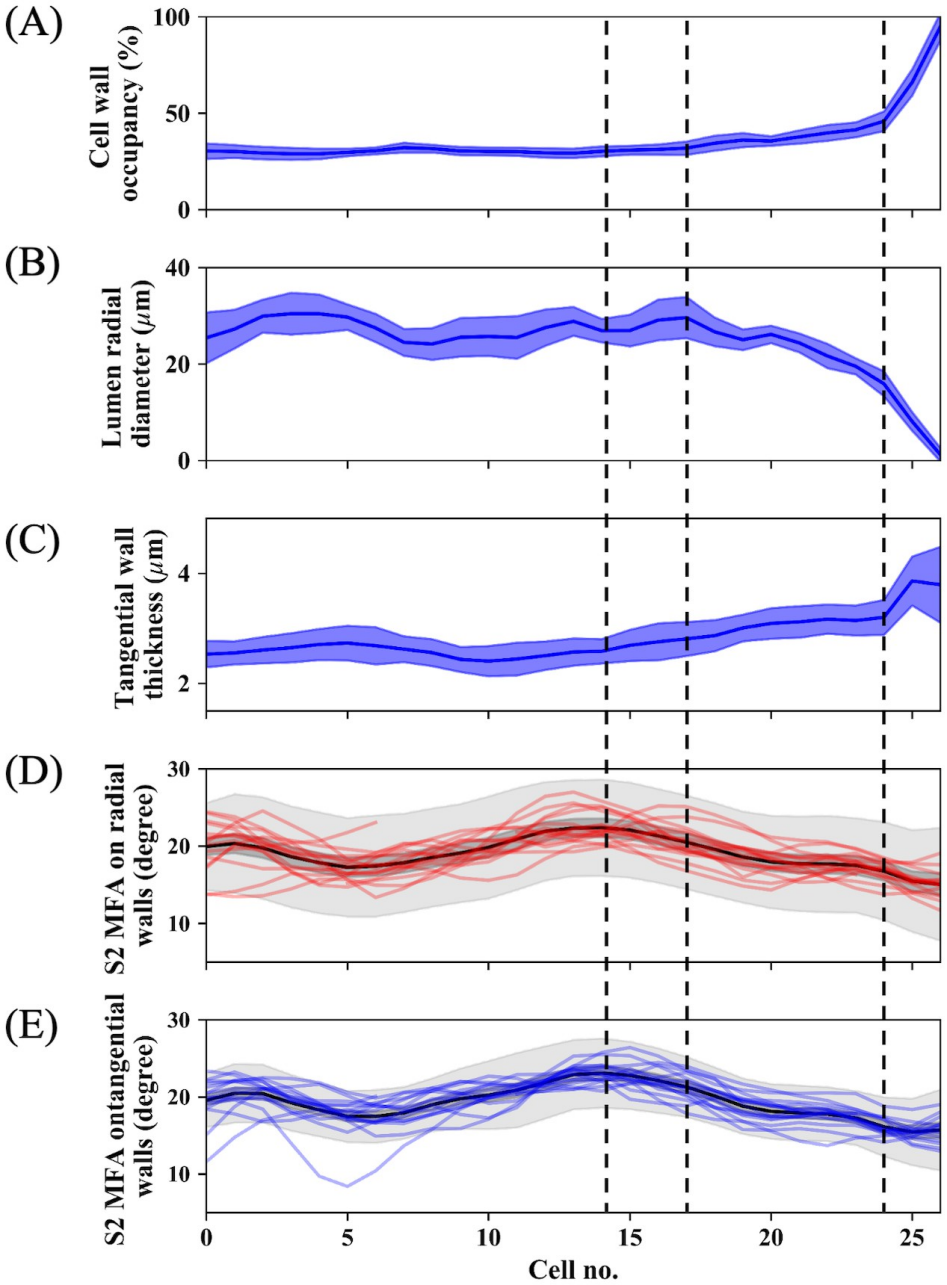
Comparison of normalized intra-annual transitional behaviors of anatomical parameters and S_2_ microfibril angle (MFA). (A) Cell wall occupancy, (B) lumen radial diameter, (C) tangential wall thickness, (D) S_2_ MFA on radial walls, and (E) S_2_ MFA on tangential walls. In (A–C), blue solid lines and the shaded area surrounding them indicate the mean intra-annual transitions and their standard deviation, respectively. In (D) and (E), black solid lines, and gray and dark gray bands correspond to the mean intra-annual trends of S_2_ MFA, their standard deviations, and standard errors of the mean, respectively. Red and blue lines in (D) and (E) indicate normalized S_2_ MFA transitions of each radial file in the radial and tangential walls, respectively. Horizontal dotted lines indicate the positions of Cell no. 15, 18, and 25 from the left side, respectively. Cell no. 1 corresponds to the tracheid positioned at the most earlywood. Cell number ranges from no. 1 to no. 27 in normalized radial files.

Fig 7 corresponds to a comparison of mean intra-annual trends of three tracheid anatomical parameters. Compared with the result of X-ray diffraction (See S3 Fig), POM results show the larger MFA values. This is partly due to the methodological difference but mainly due to the experimental imperfection in the POM experiments (See Discussion part). Ignoring absolute MFA values, the general trend obtained by each method agree with each other to some degree (earlywood MFA: 20 degree, latewood MFA: below 15 degree in POM, earlywood MFA: 14 degree, latewood MFA: 10 degree in X-ray diffraction).

The intra-annual transition in each parameter shows a strong correlation with S_2_ MFA in Table 1 (cell wall occupancy, lumen radial diameter, and tangential wall thickness) and S_2_ MFA both on radial and tangential walls. From Cell no. 1 to 14, the S_2_ MFA both on radial and tangential walls was lower when lumen radial diameter and tangential wall thickness become lower and thicker, and vice versa. At Cell no.15, the S_2_ MFA of both walls starts to decrease. At this point, lumen radial diameter is in the middle of the range observed in Cell no. 1 to 14, and tangential wall thickness was slightly increased from that of Cell no. 11. Lumen radial diameter starts to moderately decrease from Cell no. 18. At this point, cell wall occupancy simultaneously increases. Finally, radical anatomical changes occur at Cell no. 25. The S_2_ MFA of both walls continues to decrease at this point. Interestingly, the S_2_ MFA both on radial and tangential walls increases in a portion of the last tracheids (Cell no. 27). This may relate to their shorter tracheid length in addition to their thinner tangential wall [50].

**Fig 8:**
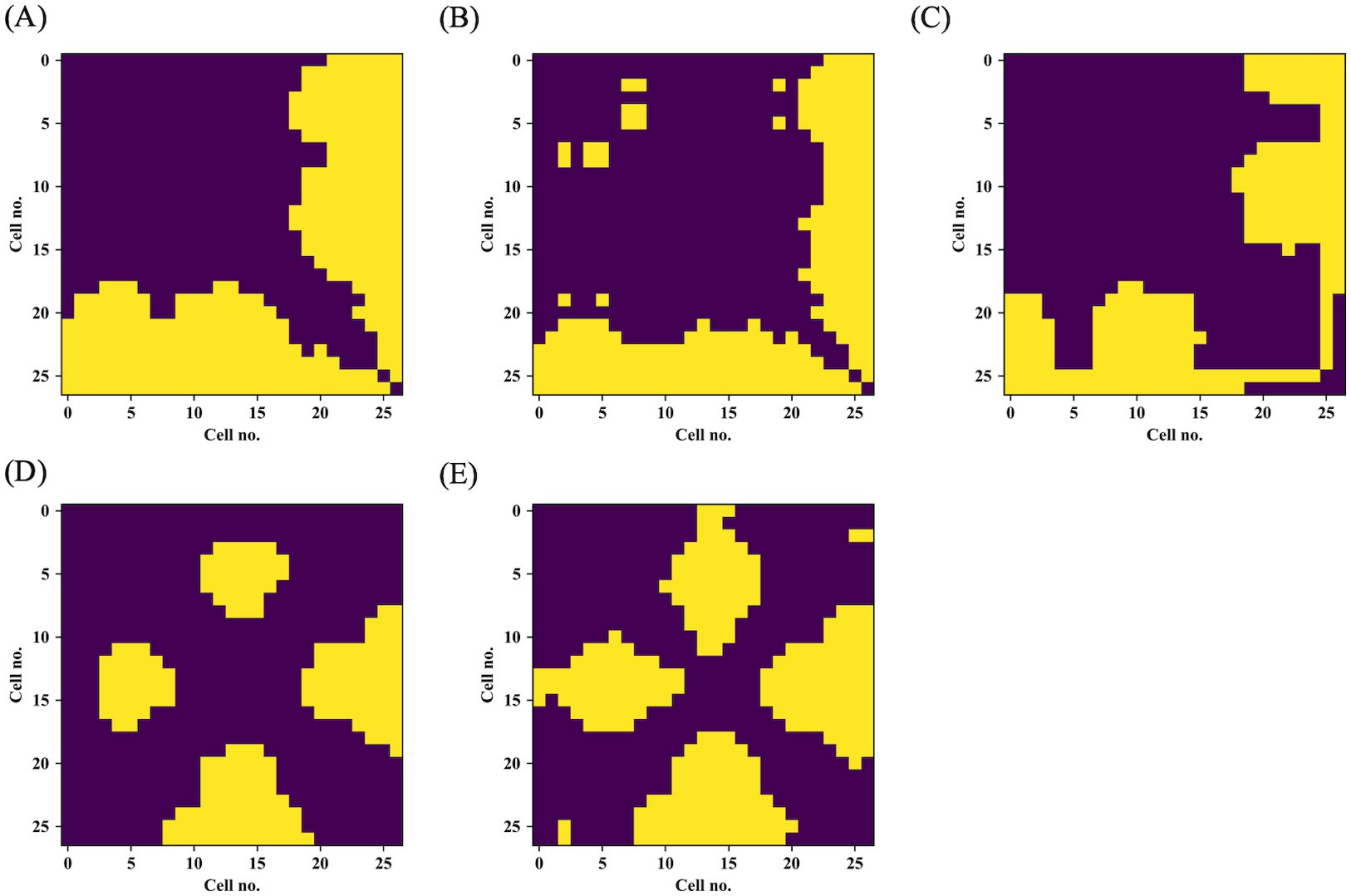
Results of Steel-Dwass test applied to anatomical parameters and S_2_ microfibril angle (MFA). (A) Cell wall occupancy, (B) lumen radial diameter, (C) tangential wall thickness, (D) S_2_ MFA on radial walls, and (E) S_2_ MFA on tangential walls. In these matrices, yellow color cells indicate the statistically significant pairs (*P* < 0.05). Observation cell number is 432.

Fig 8 shows the results of multiple comparison test among trachieds located at each Cell no. in tracehidograms. Results of Steel-Dwass test of the anatomical parameters demonstrate that large cell geometrical changes occur after Cell no. 19 and their trend continue at the end of latewood (Fig 8(A)–8(C)). On the other hand, the results obtained from S_2_ MFA on both walls express complex pattern corresponding to MFAs fluctuations in earlywood and their decrement around Cell no.15.

## Discussion

### Factors affecting intra-annual transitions on transverse tracheid dimension

According to the IAWA Committee [5], latewood formation can be characterized by two factors: decrement of tracheid radial diameter and increment of secondary cell wall thickness. Evaluation of intra-annual transitions utilizing radial files and Steel-Dwass test clearly demonstrated that the former mainly contributes the earlywood–latewood transitions rather than that of the latter in this specimen. This result agrees with previous reports [53–55]. Although a sufficient number of specimens should be treated to discuss and quantify the species-specific intra-annual transitional patterns combined with statistical tests like analysis of variance (ANOVA) or multiple comparison, utilization of radial files is a good option for tackling this topic in systematic manners.

Compared with the aforementioned parameters, tracheid tangential diameter and cell wall area seem not to affect the intra-annual transitions (see Fig 6). An almost constant cell wall area is also observed in softwood species that show different earlywood–latewood transitional behaviors and latewood ratio, such as *Abies alba*, *Picea abies*, and *Pinus sylvestris* [37]. The report [37] suggests the possibility that intra-annual tracheid morphology of these softwoods is not controlled by the degree of carbohydrate investment per tracheid but mainly by tracheid transverse dimensional changes. The present results support this hypothesis in *C*. *obtusa*. However, this hypothesis does not consider the effects of variation in intra-annual tracheid length. This point should be validated to thoroughly understand the mechanism of xylogenesis and the strategy of carbohydrate investment in softwoods using a different methodology, such as X-ray computed tomography [56].

### Relationship between tracheid morphology and S_2_ MFA

The present work demonstrated fluctuations in tracheid morphology and S_2_ MFA both on radial and tangential walls within an annual ring in a simultaneous manner as a general trend. Moderate correlations between these variables (Table 1) and their intra-annual transitions (Fig 7) suggest the possibility in this specimen that a portion of the anatomical parameters, represented by lumen radial diameter, tangential wall thickness, and cell wall occupancy, and S_2_ MFA are approximately connected with each other.

As compared to the general trend, their local fluctuation patterns and the points at which their radical changes started vary. A multiple comparison test strengthened their discrepancy. Subjective observation of the intra-annual transitions (Fig 7) and correlation coefficients (Table 1) show that intra-annual trends of S_2_ MFA on both walls resemble that of tangential wall thickness except for the last few cells (no.25-27). This result agrees with the past report that tangential wall thickness is a good indicator for MFA [4]. This point must be clarified thoroughly for a better understanding of softwood anatomical properties and their causal relationships. This can be accomplished by testing various types of softwood species with different transitional behaviors (for example, *Pinus*, *Larix*, *Tsuga* and so on) along with different methodologies, such as micro-focus synchrotron X-ray diffraction.

### Applicability and pitfalls

There are two advantageous points in analyzing softwood specimens by using experimental framework we used. First, a combination of image segmentation and automatic radial files extraction can largely lower experimental costs and will be smoothly applicable to mass data in quantitative wood anatomy or computer-aided wood identification [57]. Secondly, combination of MFA imaging and image analysis techniques enables us to systematic and semiquantitative evaluation of MFA spatial distribution cell by cell. This methodological framework will be compatible with studies on the relationship among plant hormones, tracheid morphology, and MFA [58] and for simultaneously quantifying multi-scaled anatomical transition from normal wood to compression wood.

Finally, there are three pitfalls that require attention especially in MFA imaging. The first is the transverse-section preparation step. To obtain reliable results, sections should be flat and without MFA disturbance. Disturbance of the MFA introduces critical experimental errors [59,60]. Therefore, softwood species with radical transitions and high-density hardwood species will face numerous obstacles in this step. The second is the limitation in lateral resolution of optical microscopes already mentioned in the Results section. The third pitfall is ambiguity in conversion from retardation to MFA. The conversion step requires several hyperparameters, such as section thickness and cellulose ratio in each cell wall portion. However, it is generally difficult to obtain exact values for these two parameters simultaneously when the POM experiment is conducted, so the obtained MFA values lose quantitativeness and must be treated as a semiquantitative index. If absolute MFA values are needed, they should be validated by another methodology [17].

## Conclusion

This report proposes a new microscopy and image analysis-based framework to visualize and quantitatively analyze Simultaneous acquirement of intra-annual fluctuations of anatomical parameters and S_2_ MFA was conducted for checking the validity of our framework. As a result of that, evaluation of quantitative intra-annual tracheid morphology in addition to semiquantitative MFA imaging was achieved in *C*. *obtusa*. Our experiment successfully revealed correlation between S_2_ MFA and the specific anatomical parameters. In addition, comparison of their mean intra-annual trends clearly showed their synchronized fluctuations in earlywood.

Although some technical difficulties to overcome exist, combination of all or a portion of the proposed techniques will be one of a few techniques for simultaneously quantifying tracheid anatomy and MFA in softwoods. The technique can also be applied to semi-quantitatively analyzing intra and inter-cell heterogeneity, such as evaluating the transition from normal wood to compression wood in softwoods, various cell types observed in hardwoods.

## Supporting information

S1 Fig Visualization of outlier detection in tangential wall thickness and corresponding outliers in radial files. (PDF)

S2 Fig Comparison of automatic and manual measurement of anatomical parameters. (PDF)

S3 Fig X-ray fiber diffraction diagrams obtained from the identical sample observed in this study. (PDF)

S4 Fig Normalized intra-annual transitional behaviors of a portion of the anatomical parameters. (PDF)
