# Supplementary material for: Intra-annual fluctuation in morphology and microfibril angle of tracheids revealed by novel microscopy-based imaging
Source: PLoS One. 2022 Nov 15;17(11):e0277616. doi: 10.1371/journal.pone.0277616 (PMC9665381; doi:10.1371/journal.pone.0277616)
Supplement: S1 Fig — (PDF) [file pone.0277616.s001.pdf]

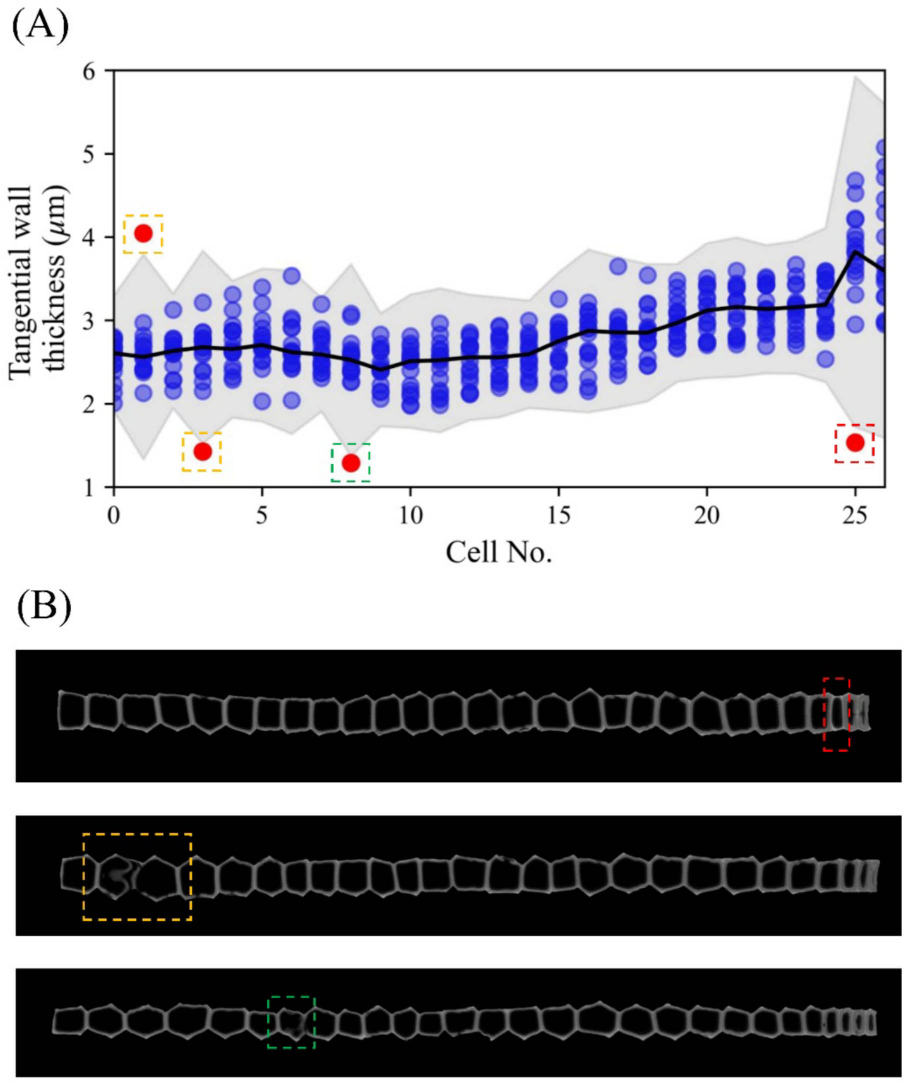

S1 Fig. Visualization of outlier detection in tangential wall thickness and corresponding outliers in radial files. (A) Tangential wall thickness measurements for each cell number. Red dots indicate detected outliers. (B) Examples of radial files containing outliers. A red dotted rectangle in (A) corresponds to a resin cell in (B). Orange and green dotted rectangles in (A) correspond to ruptured cells in (B).
