# Supplementary material for: Intra-annual fluctuation in morphology and microfibril angle of tracheids revealed by novel microscopy-based imaging
Source: PLoS One. 2022 Nov 15;17(11):e0277616. doi: 10.1371/journal.pone.0277616 (PMC9665381; doi:10.1371/journal.pone.0277616)
Supplement: S2 Fig — (PDF) [file pone.0277616.s002.pdf]

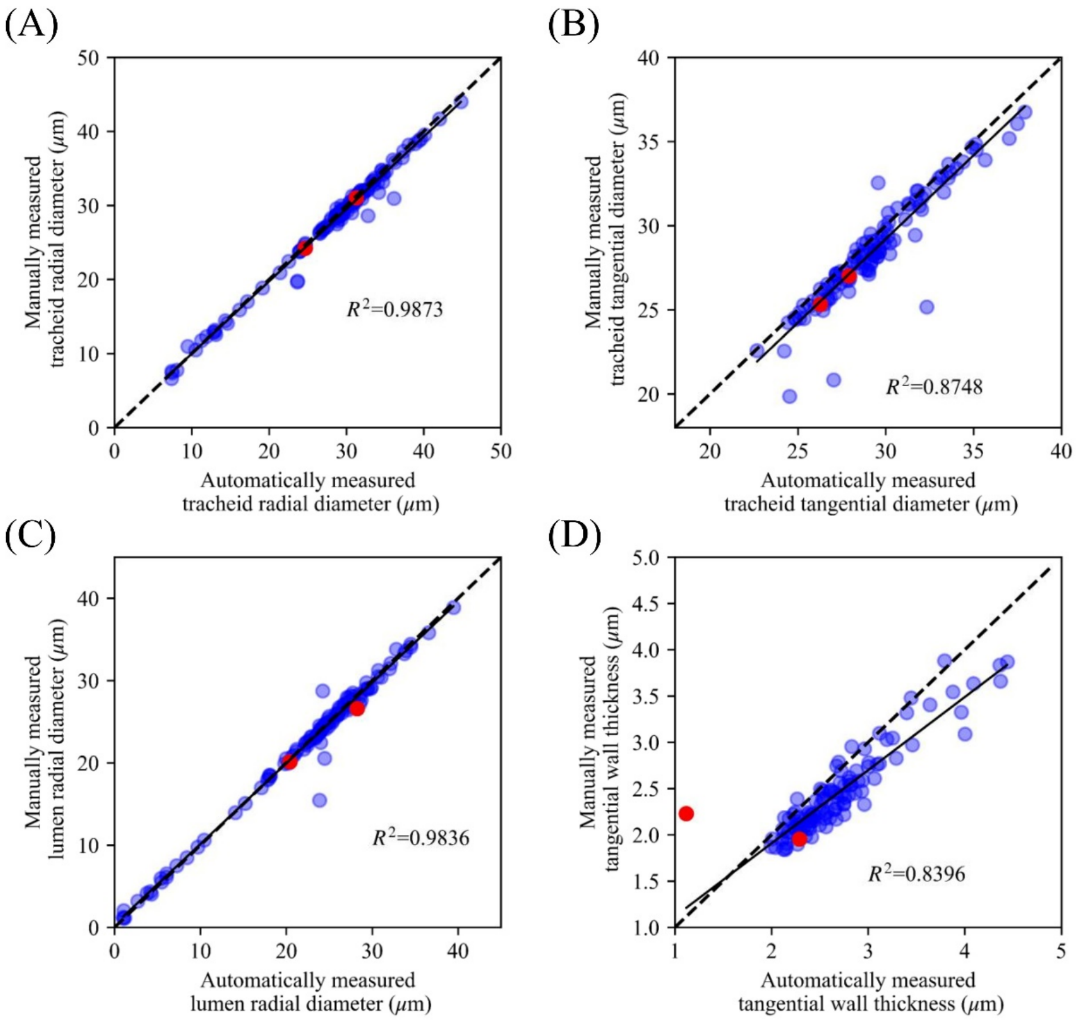

S2 Fig. Comparison of automatic and manual measurement of anatomical parameters. (A) Tracheid radial diameter, (B) tracheid tangential diameter, (C) lumen radial diameter, and (D) tangential wall thickness. Red dots indicate ruptured cells. Black lines and R^2 values in each figure indicate regression lines and their coefficients of determination obtained from automatic vs. manual measurements. Black dotted lines correspond to those with slopes equal to 1.
