# Supplementary material for: Intra-annual fluctuation in morphology and microfibril angle of tracheids revealed by novel microscopy-based imaging
Source: PLoS One. 2022 Nov 15;17(11):e0277616. doi: 10.1371/journal.pone.0277616 (PMC9665381; doi:10.1371/journal.pone.0277616)
Supplement: S3 Fig — (PDF) [file pone.0277616.s003.pdf]

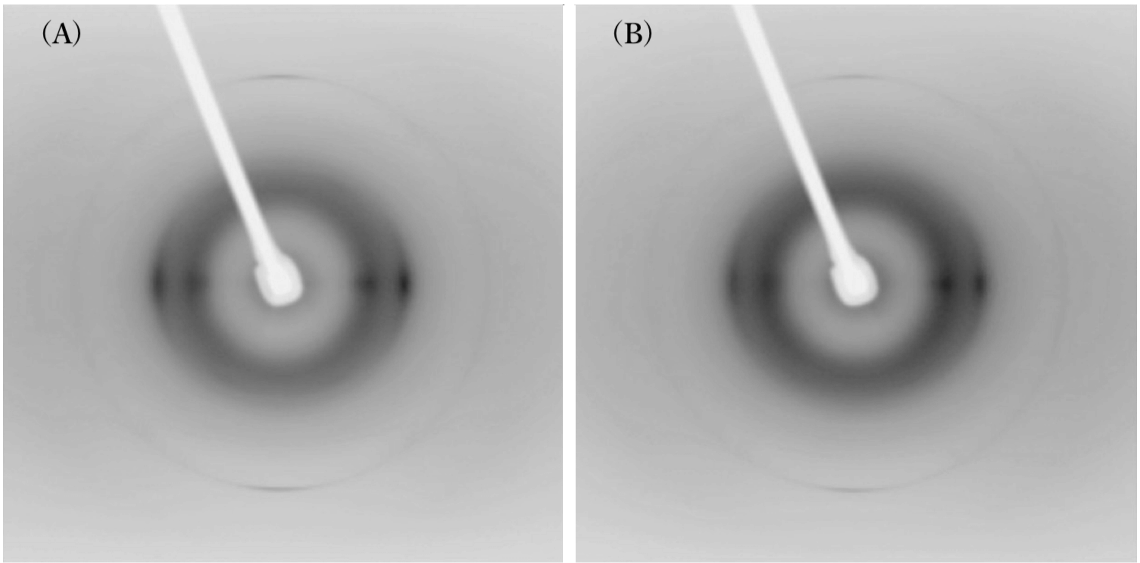

S3 Fig. X-ray fiber diffraction diagrams obtained from the identical sample observed in this study. The halo comes from non-crystalline scattering from epoxy resin used as embedding medium for sectioning. X-way was collimated to 0.3 mm diameter to obtain earlywood (A) and latewood (B) data separately. Azimuthal intensity profiles of (200) diffraction, after subtracting the background halo, were analysed by conventional Cave method. 14 and 10 degrees were obtained as average values of MFA in earlywood and latewood, respectively.
