# Supplementary material for: Intra-annual fluctuation in morphology and microfibril angle of tracheids revealed by novel microscopy-based imaging
Source: PLoS One. 2022 Nov 15;17(11):e0277616. doi: 10.1371/journal.pone.0277616 (PMC9665381; doi:10.1371/journal.pone.0277616)
Supplement: S4 Fig — (PDF) [file pone.0277616.s004.pdf]

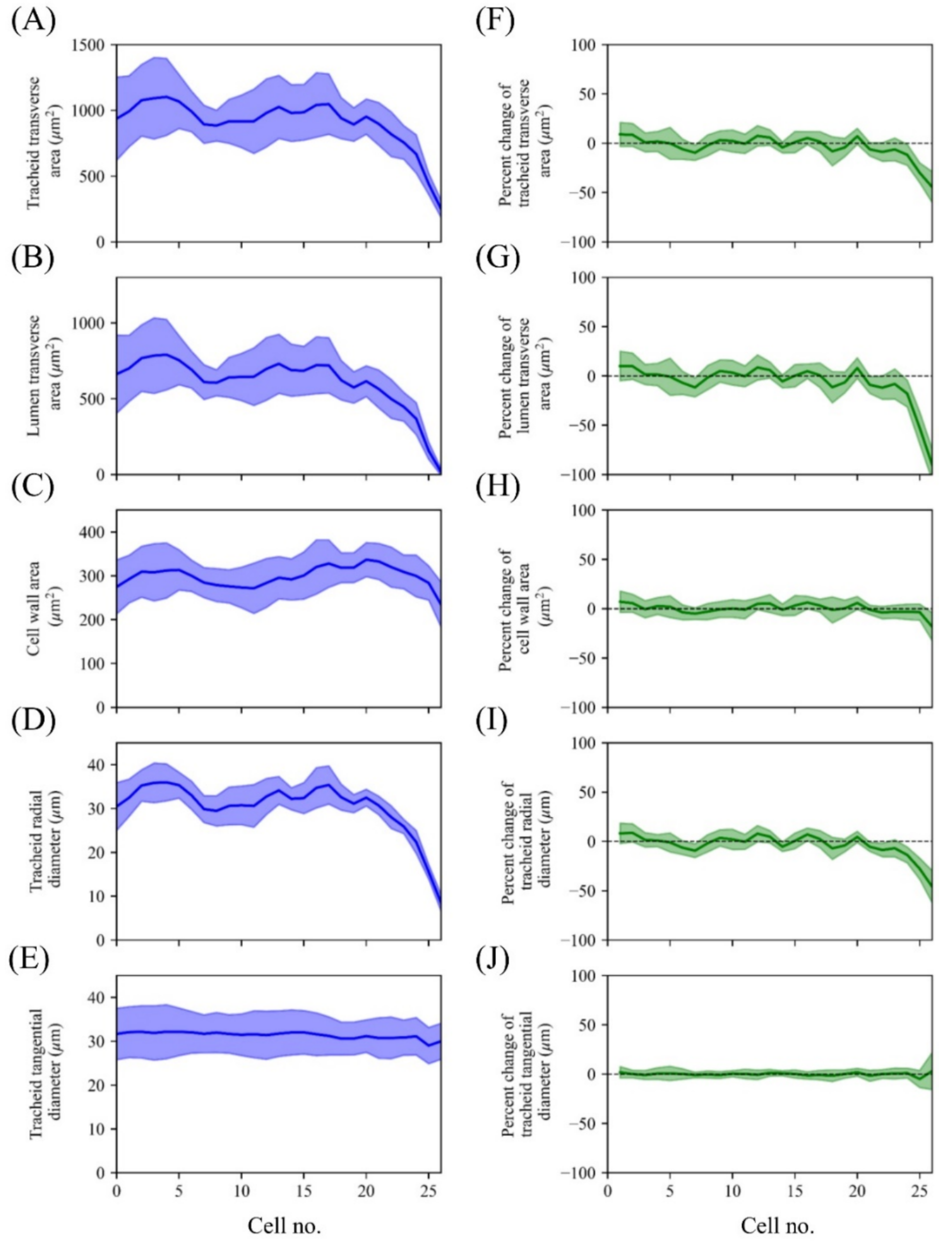

S3 Fig. Normalized intra-annual transitional behaviors of a portion of the anatomical parameters. (A) Tracheid transverse area, (B) lumen transverse area, (C) cell wall area, (D) tracheid radial diameter and (E) tracheid tangential diameter. (F–J) Percentage changes in each anatomical parameter (A–E) from preceding tracheids. Solid lines and the shaded area surrounding them indicate the mean intra-annual transitions and their standard deviations, respectively. Cell no. 0 corresponds to the tracheid positioned at the most earlywood side. Cell number ranges from no. 0 to no. 26 in normalized radial files.
